# Bilateral Localized-Tenosynovial Giant Cell Tumor of the Knee: Case Report and Review

**DOI:** 10.3390/jcm15031016

**Published:** 2026-01-27

**Authors:** Vasiliki D. Dania, Dimitra P. Papagelopoulos, Ioannis Tolis, Maria Papanikolaou, Nikolaos A. Stavropoulos, Olympia Papakonstantinou, Penelope Korkolopoulou, Panayiotis J. Papagelopoulos

**Affiliations:** 1 First Department of Orthopedic Surgery, School of Medicine, National and Kapodistrian University of Athens, Attikon University General Hospital, 12462 Athens, Greecegiannistolis75@gmail.com (I.T.);; 2 First Department of Pathology, Medical School, National and Kapodistrian University of Athens, Laikon General Hospital, 11527 Athens, Greece; 3 Second Department of Radiology, School of Medicine, National and Kapodistrian University of Athens, Attikon General University Hospital, 12462 Athens, Greece

**Keywords:** TGCT, tenosynovial giant cell tumor, bilateral knee, PVNS, pigmented villonodular tenosynovitis

## Abstract

**Background/Objectives:** Tenosynovial giant cell tumor (TGCT), formerly known as pigmented villonodular tenosynovitis (PVNS), is a rare, benign, inflammatory mesenchymal neoplasm originating from the synovium of joints, bursae, or tendon sheaths. Although TGCT can affect any joint, the knee is the most commonly involved site, particularly in cases of diffuse-type TGCT. Bifocal or multifocal involvement is exceedingly uncommon. **Methods:** Herein, we present a case of localized TGCT with bilateral knee involvement in a 48-year-old female. **Results:** The patient underwent open arthrotomy with marginal excision of the localized lesions in both knees. Histology and immunohistochemistry staining conformed the diagnosis. At the five-year follow-up, the patient remains asymptomatic and free of recurrence. **Conclusions:** Given the rarity of bilateral TGCT, clinicians should maintain a high index of suspicion when evaluating patients presenting with bilateral knee pain and swelling and include TGCT in the differential diagnosis. To our knowledge, this represents the fifteenth reported case of bilateral knee TGCT in the literature.

## 1. Introduction

Tenosynovial giant cell tumor (TGCT) is an uncommon, benign, inflammatory mesenchymal neoplasm arising from the synovium of joints, bursae, or tendon sheaths [1,2]. TGCT is traditionally divided into two subtypes: localized TGCT and diffuse TGCT [3]. However, at an international consensus meeting held in Germany in 2022, the classification was refined into nodular TGCT (N-TGCT)—corresponding to the localized type—and diffuse TGCT (D-TGCT) [4]. According to the 2013 World Health Organization (WHO) classification, the previously distinct terms *pigmented villonodular synovitis* (PVNS) and *giant cell tumor of the tendon sheath* (GCTTS) were unified under a single nomenclature: *Tenosynovial Giant Cell Tumor* [1].

Localized TGCT typically presents as a solitary, lobulated lesion originating from the tendon sheath and, less frequently, from the synovial lining of a joint. The localized type most commonly affects small joints—such as the digits of the hands and feet, or the wrist—and less frequently large joints like the knee. The incidence rate of N-TGCT in the extremities is approximately 11 per million person-years [5,6,7].

In contrast, diffuse TGCT exhibits a more aggressive and locally destructive behavior, involving a large portion or the entirety of the synovium and presenting with a multinodular appearance. The reported incidence of D-TGCT is approximately 5–8.4 per million person-years, with the knee being the most frequently affected joint, followed by the ankle and hip [5,6,7].

TGCT—regardless of subtype—most commonly affects a single joint. Bilateral, bifocal, or multifocal joint involvement is exceedingly rare. Herein, we present a case of bilateral diffuse-type TGCT of the knees in a 48-year-old female, representing the fifteenth reported case of bilateral knee TGCT in the literature. In light of this rare presentation, we discuss relevant diagnostic challenges and treatment considerations.

## 2. Case Presentation

A 48-year-old woman was referred to our hospital in July 2021 with progressively worsening intermittent pain and swelling in both knees. The symptoms had begun approximately one year earlier and were contemporaneous in onset. There was no history of trauma or concomitant diseases, and her past medical history was unremarkable. Despite the conservative treatment with the use of non-steroidal anti-inflammatory drugs (NSAIDs) and several sessions of physiotherapy, the pain and swelling in both knees persisted and gradually worsened.

On physical examination, tenderness was noted in the suprapatellar region extending from the patellar tendon to the anterolateral joint line. Both knees were swollen, and the range of motion was restricted between 0° and 145°. The McMurray test was negative.

**Figure 1:**
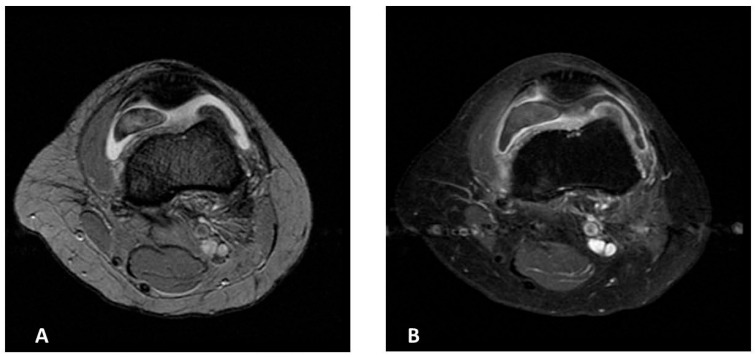
MR images of the left knee (**A**,**B**). (**A**) Axial T2-weighted MR image of the suprapatellar pouch of the left knee showing intraarticular lesion with cloud-like dark areas due to hemosiderin deposition. (**B**) Post-contrast T1 fat-saturated MR image of the same region demonstrating synovitis and enhancement of synovial proliferations.

Magnetic Resonance Imaging (MRI) of the left knee demonstrated mild joint effusion, synovial hyperplasia, and a mass-like lesion in continuity with the synovium in the anteromedial patellofemoral joint space, measuring approximately 40 × 26 × 11 mm. The lesion showed homogeneous contrast enhancement and a characteristic “blooming” artifact on gradient-echo sequences. It appeared hypointense on T2-weighted images, consistent with hemosiderin deposition, and iso- to hypointense on T1-weighted images—findings suggestive of localized TGCT (Figure 1A,B).

**Figure 2:**
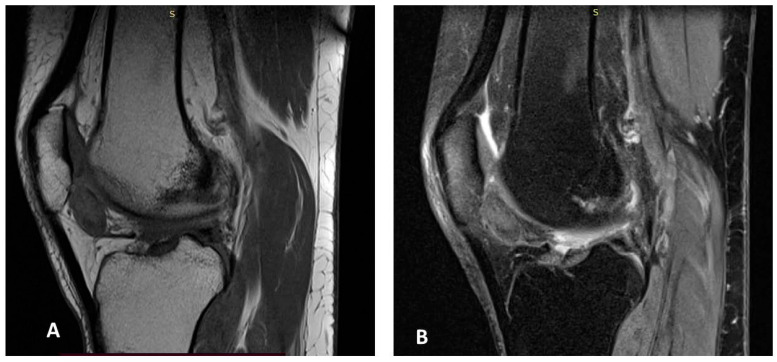
MR images of the right knee (**A**,**B**). Sagittal T1-weighted (**A**) and gradient-echo (**B**) MR images of the right knee showing an intraarticular soft-tissue mass with foci of low signal intensity in the anterior compartment, consistent with hemosiderin deposition.

MRI of the right knee revealed a similar mass-like lesion in the posterior compartment, measuring 19 × 11 × 20 mm, accompanied by mild joint effusion. The signal characteristics on both T2 fat-saturated and T1-weighted images mirrored those of the left knee (Figure 2A,B).

**Figure 3:**
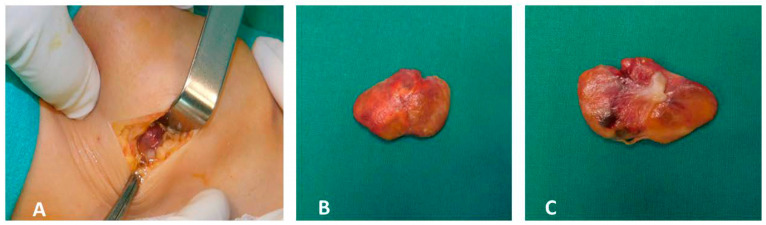
Intraoperative photographs (**A**–**C**). Intraoperative findings following open arthrotomy and marginal resection of the localized lesions in both knees. The excised specimens exhibit a yellow-brown coloration and irregular, lobulated surface.

The patient underwent open arthrotomy with marginal excision of the localized lesions in both knees. Grossly, the excised specimens were yellow-brown in color and exhibited an irregular, lobulated surface (Figure 3A–C).

**Figure 4:**
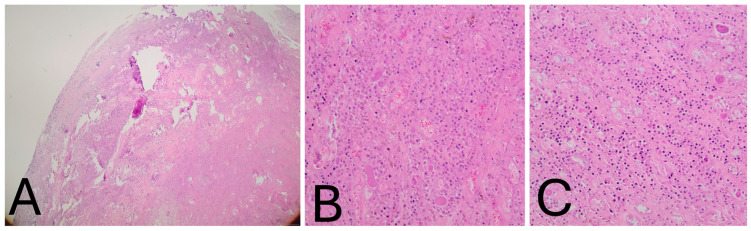
(**A**–**C**). Histological examination. (**A**) Low-power view showing the well-circumscribed tumor margins (H&E, ×4). (**B**) High magnification view demonstrating mononuclear histiocyte-like and epithelioid cells containing peripheral hemosiderin granules, interspersed with foamy macro-phages within abundant collagenous stroma. Osteoclast-like giant cells are visible in the upper right field (H&E, ×200). (**C**) Additional high-power field highlighting multinucleated giant cells (H&E, ×200).

**Figure 5:**
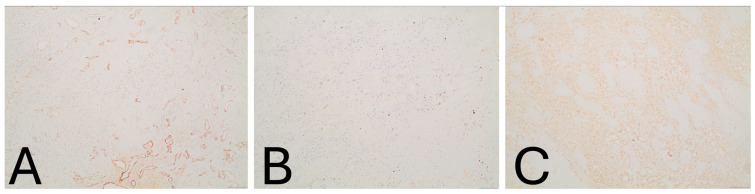
Immunohistochemistry (IHC) staining (**A**–**C**). Immunohistochemistry (IHC) staining of tumor cells showing negativity for (**A**) SMA (×100). (**Β**) Low proliferative activity with Ki-67 labeling index of approximately 1% (×100). (**C**) Diffuse cytoplasmic immunoreactivity of tumor cells for PGM-1 (×100).

**Figure 6:**
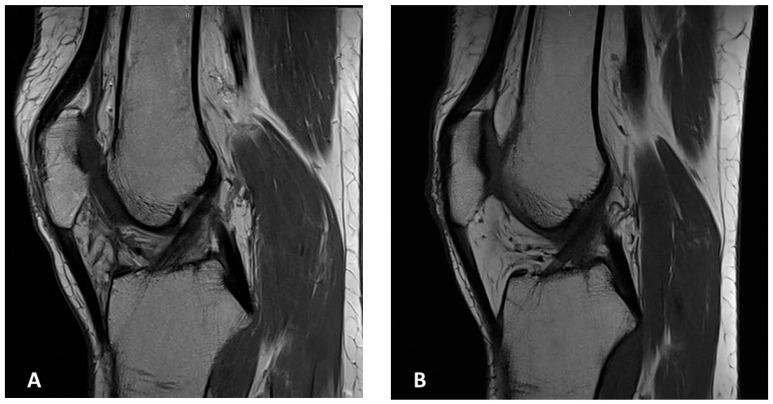
MR images of both knees. Sagittal MR images of the right (**A**) and left (**B**) knee at the latest follow-up showing no evidence of local recurrence.

Histological Findings: Microscopically, the tumor was lobulated, well-circumscribed, and composed of sheets of neoplastic mononuclear cells admixed with multinucleated osteoclast-like giant cells and scattered inflammatory cells, including foamy macrophages. The stroma was collagenous. The mononuclear cells consisted of smaller histiocyte-like cells with pale cytoplasm and round nuclei, and larger epithelioid cells with amphophilic cytoplasm and vesicular nuclei. Hemosiderin granules were frequently observed within the cytoplasm (Figure 4A–C). Mitotic activity was low, and no areas of high-grade morphology—such as nuclear atypia, pleomorphism, increased cellularity, elevated mitotic count, or necrosis—were identified. Immunohistochemistry revealed that the histiocyte-like cells were positive for PGM-1 and negative for SOX-10, S-100, SMA, and CD34 (Figure 5A–C). The morphological and immunohistochemical findings were consistent with the diagnosis of localized tenosynovial giant cell tumor. At the five-year follow-up, the patient remains asymptomatic and free of recurrence (Figure 6A,B). Institutional Review Board Approval was obtained for this study (ΕΒΔ 599/8-11-2021).

## 3. Discussion

The first documented case of tenosynovial giant cell tumor (TGCT) was reported by Chassaignac in 1852, describing involvement of the flexor tendons of the fingers [8]. Simon subsequently defined the localized type in 1865 [9], and Moser, in 1909, described the diffuse form of the disease affecting the knee [10]. In 1941, Jaffe et al. presented 12 cases, outlining the pathological, radiological, and clinical features of TGCT and unifying the various forms of the disease (focal, nodular, diffuse, intra- and extra-articular) under a single entity, proposing a reactive or inflammatory origin [11]. Granowitz et al. later classified TGCT into two distinct clinical forms—localized and diffuse—in 1976 [3]. More recently, the 2022 International Consensus Meeting in Germany redefined the terminology as nodular TGCT (N-TGCT), corresponding to the localized form, and diffuse TGCT (D-TGCT) [4].

Historically, several terms have been used to describe this tumor, including *synovial xanthoma*, *synovial fibroendothelioma*, *chronic hemorrhagic villous synovitis*, *fibrohaemosideric sarcoma*, and *fibrous xanthoma of the synovial membrane* [11,12,13,14].

In 2006, West et al. demonstrated the neoplastic nature of TGCT by identifying chromosomal abnormalities, including trisomy of chromosomes 5 and 7 and translocations involving 1p11–13 with 2q37 [15]. The translocation t(1;2)(p13;q37) results in overexpression of the colony-stimulating factor 1 (CSF1) gene through formation of a COL6A3–CSF1 fusion product, which occurs in approximately 2–16% of tumor cells. These neoplastic cells overexpress CSF1, promoting tumor growth via an autocrine loop and recruitment of non-neoplastic cells through a paracrine “landscape effect” [15,16,17].

Macroscopically, N-TGCT typically presents as a well-circumscribed, encapsulated or pedunculated lesion measuring 0.5–4 cm, while D-TGCT exhibits a villous (intra-articular) or multinodular (extra-articular) pattern, often exceeding 5 cm and infiltrating a large portion or the entirety of the synovium. Microscopically, both subtypes share similar histopathological features, including mononuclear histiocyte-like cells, siderophages (macrophages containing hemosiderin granules), multinucleated giant cells, foamy macrophages, fibroblast-like synoviocytes, stromal hyalinization, and a variable lymphocytic infiltrate [1,2,18,19,20,21,22].

Plain radiographs, typically performed as the first-line imaging modality, may appear normal or demonstrate nonspecific findings such as soft tissue swelling, joint effusion, or well-defined bone erosions with sclerotic margins due to cortical pressure effects. Bone mineralization and joint space are usually preserved, and calcification is absent [12,23,24,25]. Radiography remains useful for excluding other entities such as degenerative disease, synovial chondromatosis, or aggressive neoplasms [12].

Magnetic resonance imaging (MRI) is the modality of choice for evaluating TGCT. In D-TGCT, MRI reveals multinodular synovial thickening with low-to-intermediate signal intensity on both T1- and T2-weighted images, and a characteristic “blooming” artifact on gradient-echo sequences due to hemosiderin deposition—a nearly pathognomonic finding [12,26,27]. In N-TGCT, MRI demonstrates a well-defined mass with similar signal characteristics, though blooming is less prominent [12].

Clinical presentation depends on lesion location and disease extent but commonly includes joint pain, swelling, stiffness, locking, and restricted range of motion [28]. According to Mastboom et al., both TGCT subtypes show a predilection for the knee joint (46% for N-TGCT and 64% for D-TGCT), followed by the hand/wrist (localized form) and ankle/hip (diffuse form) [5]. Van der Heijden et al. further reported that the knee is involved in approximately 75% of D-TGCT cases [29]. TGCT typically affects adults aged 30–50 years, with recurrence rates ranging from 9 to 14% for localized TGCT and 23–72% for diffuse TGCT, the latter being 2.6 times higher [1,27,30].

TGCT is predominantly monoarticular; bilateral or multifocal disease is exceedingly rare. The present case represents the fifteenth reported instance of bilateral knee TGCT.

Historically, the first bilateral knee case was described by Kelikian and Lewis (1949) in a 15 year-old girl and her brother, both with diffuse TGCT and possible familial predisposition [31]. Subsequent bilateral knee cases include those reported by Greenfield and Wallace (1950) [32], Gehweiler and Wilson (1969) [33], Sharma et al. (2009) [34], Kim et al. (2010) [35], Soubai et al. (2010) [36], Cho et al. (2012) [37], Klammer et al. (2013) [38], Shah et al. (2015) [39], Meftah et al. (2016) [40], Fernandes et al. (2018) [41], Okamura et al. (2022) [42], and Lachkar et al. (2024) [43] (Table 1).

Bilateral TGCT has also been described in other joints, including the shoulders [44], wrists [45], thumbs [46], hips [47,48,49], ankles [22,50,51], and Achilles tendons [52,53]. Multifocal or bifocal presentations have been rarely documented.

Surgical excision remains the gold standard for TGCT management, using either open or arthroscopic techniques depending on the disease extent. In extra-articular N-TGCT, complete excision usually results in low recurrence rates [29]. For intra-articular N-TGCT of the knee, open surgery appears superior to arthroscopy: Mastboom et al. reported recurrence rates of 9% after open excision versus 18% following arthroscopic removal [54]. In D-TGCT, van der Heijden et al. noted recurrence rates of 14% after open versus 40% after arthroscopic synovectomy [29].

Adjuvant radiotherapy, including external beam radiation (EBR) and radiosynoviorthesis (RSO), has been employed for extensive or recurrent disease, although its use remains controversial due to risks of infection, radionecrosis, and secondary malignancy [55,56].

Molecular advances identifying the CSF1–CSF1R signaling pathway as central to TGCT pathogenesis have led to the development of targeted therapies. Pexidartinib, a selective CSF1R inhibitor, is the first FDA-approved systemic therapy for advanced or unresectable TGCT [57,58,59,60]. Other investigational or selective inhibitors include emactuzumab, cabiralizumab, lacnotuzumab, and sotuletinib [61,62,63,64,65,66], while non-selective inhibitors such as nilotinib, imatinib, and pimicotinib have shown variable efficacy [67,68,69,70]. Most recently, the FDA accepted a New Drug Application (NDA) for vimseltinib, another selective CSF1R inhibitor, offering a promising therapeutic option for patients with TGCT [71,72].

## 4. Conclusions

Tenosynovial giant cell tumor (TGCT) is a rare, benign, and typically monoarticular neoplasm. Among patients with diffuse TGCT (D-TGCT), the knee joint is the most frequently affected site. Bilateral TGCT—either diffuse or localized—is exceptionally uncommon, with only 14 cases of bilateral knee involvement previously reported in the literature. The present case represents the fifteenth such report and the fifth involving bilateral diffuse-type TGCT of the knees. Our patient exhibited classic clinical, radiologic, and histopathologic features of D-TGCT in both knees. Surgical excision remains the gold standard of treatment, and the choice between open and arthroscopic approaches should be guided by the lesion’s extent and anatomical location. Given the rarity of bilateral TGCT, clinicians should maintain a high index of suspicion when evaluating patients presenting with bilateral knee pain and swelling and include TGCT in the differential diagnosis.

## Figures and Tables

**Table 1:** Bilateral tenosynovial giant cell tumor (TGCT) cases in the literature.

	No	Age/Sex	Type of TGCT	Surgical Procedure	FU (Months)	Recurrence
1	(Kelikian H et al., 1949) [31]	15/F	Diffuse	Synovectomy	N/A	N/A
2	(Kelikian H et al., 1949) [31]	young/M	Diffuse	Synovectomy	N/A	N/A
3	(Greenfield MM et al., 1950) [32]	24/M	Diffuse	Exploratory arthrotomy/Irradiation	8	N/A
4	(Gehweiler JA et al., 1969) [33]	56/F	Diffuse	Synovectomy patellectomy, meniscectomy	24 (Left Knee) N/A (Right Knee)	No for the Left Knee N/A (Right Knee)
5	(Sharma H et al., 2009) [34]	58/F	Localized (anterior compartment)	Partial synovectomy-open excisional	12	Only Stiff knee treated with manipulation under anesthesia
6	(Kim HS et al., 2010) [35]	29/M	Localized	Open mass excision	N/A	N/A
7	(Soubai et al., 2011) [36]	20/F	Diffuse	Open total synovectomy	N/A	N/A
8	(Cho et al., 2012) [37]	16/M	Localized	Arthroscopic excision	14	No Recurrence
9	(Klammer G et al., 2013) [38]	16/F	Diffuse	Arthroscopic Total Synovectomy and open dorsal synovectomy and open popliteal	18	42 months (Left Knee) 48 months (Right Knee)
10	(Shat SH et al., 2015) [39]	62/M	Diffuse	Arthroscopic excision with synovectomy and chondroplasty	6 years	At 2 years for the Right Knee
11	(Meftah A et al., 2016) [40]	28/	Diffuse	Total synovectomy	N/A	N/A
12	(Fernandes TL et al., 2018) [41]	28/F	Localized	Synovectomy	N/A	N/A
13	(Okamura H et al., 2022) [42]	28/F	Localized	Arthroscopic resection	6	No recurrence
14	(Lachkar A et al., 2024) [43]	20/F	Diffuse	Open total synovectomy	12	No recurrence
15	(Present case, 2025)	48/F	Localized	Open excision	60	No recurrence

## Data Availability

The original contributions presented in this study are included in the article. Further inquiries can be directed to the corresponding author.
